# Parameterization of a single H-bond in Orange Carotenoid Protein by atomic mutation reveals principles of evolutionary design of complex chemical photosystems

**DOI:** 10.3389/fmolb.2023.1072606

**Published:** 2023-01-26

**Authors:** Marcus Moldenhauer, Hsueh-Wei Tseng, Anastasia Kraskov, Neslihan N. Tavraz, Igor A. Yaroshevich, Peter Hildebrandt, Nikolai N. Sluchanko, Georg A. Hochberg, Lars-Oliver Essen, Nediljko Budisa, Lukas Korf, Eugene G. Maksimov, Thomas Friedrich

**Affiliations:** ^1^ Department of Bioenergetics, Institute of Chemistry PC 14, Technische Universität Berlin, Berlin, Germany; ^2^ Department of Biocatalysis, Institute of Chemistry L1, Technische Universität Berlin, Berlin, Germany; ^3^ Department of Biophysics, Faculty of Biology, Lomonosov Moscow State University, Moscow, Russia; ^4^ A.N. Bach Institute of Biochemistry, Federal Research Center Fundamentals of Biotechnology of Russian Academy of Sciences, Moscow, Russia; ^5^ Max-Planck-Institute of Terrestrial Microbiology, Evolutionary Biochemistry Group, Marburg, Germany; ^6^ Department of Chemistry and Unit for Structural Biology, Philipps-Universität Marburg, Marburg, Germany; ^7^ Department of Chemistry, University of Manitoba, Winnipeg, MB, Canada

**Keywords:** atomic mutations, hydrogen bond strength/energy, non-canonical amino acids, orthogonal translation, Orange Carotenoid Protein

## Abstract

**Introduction:** Dissecting the intricate networks of covalent and non-covalent interactions that stabilize complex protein structures is notoriously difficult and requires subtle atomic-level exchanges to precisely affect local chemical functionality. The function of the Orange Carotenoid Protein (OCP), a light-driven photoswitch involved in cyanobacterial photoprotection, depends strongly on two H-bonds between the 4-ketolated xanthophyll cofactor and two highly conserved residues in the C-terminal domain (Trp288 and Tyr201).

**Method:** By orthogonal translation, we replaced Trp288 in *Synechocystis* OCP with 3-benzothienyl-*L*-alanine (BTA), thereby exchanging the imino nitrogen for a sulphur atom.

**Results:** Although the high-resolution (1.8 Å) crystal structure of the fully photoactive OCP-W288_BTA protein showed perfect isomorphism to the native structure, the spectroscopic and kinetic properties changed distinctly. We accurately parameterized the effects of the absence of a single H-bond on the spectroscopic and thermodynamic properties of OCP photoconversion and reveal general principles underlying the design of photoreceptors by natural evolution.

**Discussion:** Such “molecular surgery” is superior over trial-and-error methods in hypothesis-driven research of complex chemical systems.

## 1 Introduction

**FIGURE 1:**
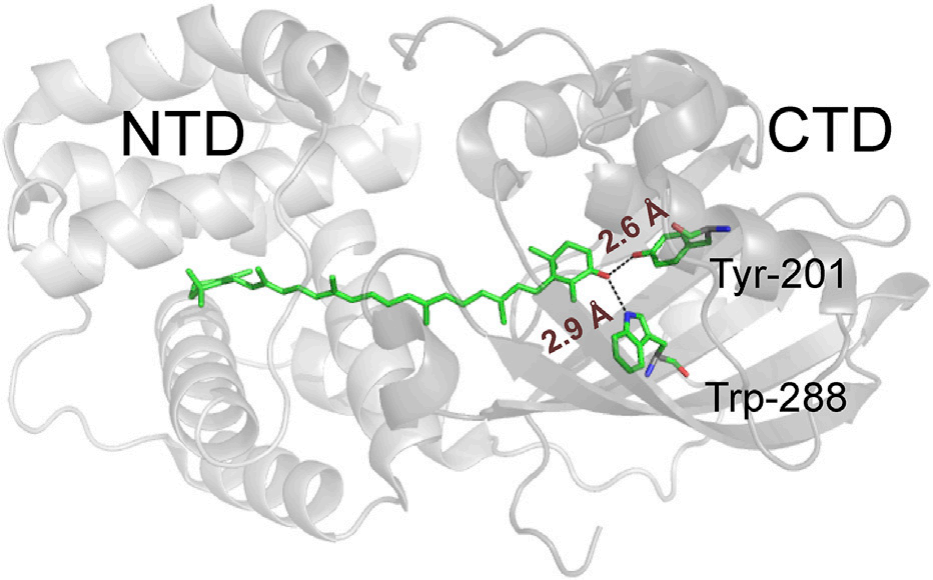
Structural representation of wild-type *Synechocystis* OCP with the N- and C-terminal domains depicted in different shades of grey. The critical H-bond-donating residues (Trp288 and Tyr201) and the ECN cofactor are shown as ball-and-stick models, while the H-bonds are indicated by dashed lines.

Maintaining the marginally stable three-dimensional architecture of proteins often requires a complicated network of weak interactions, including hydrogen bonds. The study of individual hydrogen bonds in the context of such complex protein structures is often a formidable task. A kind of “molecular surgery” (Thi To et al., 2022) with “atomic mutations” (Minks et al., 1999) is required to remove or replace individual atoms or groups of atoms in the context of complex proteins containing thousands of atoms with a complicated network of, for example, covalent, non-covalent or hydrogen bonding and van der Waals interactions. It has been suggested that the function of the Orange Carotenoid Protein (OCP), a blue light-driven photoswitch involved in photoprotection of cyanobacteria (Holt and Krogmann, 1981), strongly depends on two critical H-bonds between the ketocarotenoid cofactor and a tryptophan (Trp) and a tyrosine (Tyr) residue in the surrounding protein matrix (Figure 1). However, classical protein mutagenesis does not allow the precise parameterization of the contributions of these H-bonds to a protein’s functional integrity.

Structurally, the 35 kDa OCP consists of an N-terminal domain (NTD) composed entirely of α-helices and a mixed α/β-type C-terminal domain (CTD) of about equal size, both encapsulating a single ketocarotenoid molecule in a common central cavity (Kerfeld et al., 2003). Upon absorption of a photon, OCP converts from the dark-adapted, orange OCP^O^ state to the red, active OCP^R^ state. The embedded ketocarotenoid is at the heart of OCP photoswitching activity and function. In native cyanobacteria this cofactor is 3'-hydroxyechinenone (Muzzopappa and Kirilovsky, 2020); whereas the chemical identity in the equally active recombinant form is a 4-ketolated xanthophyll. In the compact OCP^O^ state, for which X-ray crystal structures are available (e.g., PDB codes 3MG1, 4XB5, 5UI2), the 4-keto oxygen atom of the xanthophyll is involved in an H-bonding network to the two highly conserved residues within the C-terminal domain (Tyr201 and Trp288 according to amino acid numbering of *Synechocystis* OCP). This local network fixes the cofactor in a rigid, slightly bent and twisted conformation (Figure 1) (Kerfeld et al., 2003; Leverenz et al., 2015). The H-bonds in this network break upon photoexcitation, releasing the terminal β-ring of the xanthophyll, leading to a number of detectable spectroscopic and conformational changes, e.g., xanthophyll isomerization and translocation, separation of domains, and a red-shift in absorbance (Leverenz et al., 2015).

The structure of the OCP^R^ state has not yet been elucidated because it is very elongated and flexible (Gupta et al., 2015; Sluchanko et al., 2017a). In OCP^R^, the protein is able to interact closely with the water-soluble antenna complexes of cyanobacteria, the phycobilisomes (PBSs). By exposing the chromophore to the terminally emitting pigments of the PBSs, OCP^R^ acts as an efficient quencher of PBS fluorescence, protecting the underlying photosystems from the deleterious effects of overexcitation and dissipating the electronic excitation energy into heat.

Mutations of Tyr201 and Trp288 often destabilize the compact OCP^O^ form and generate permanently red-shifted OCP forms with separated domains that are - despite not being photoactive - constitutively capable of PBS fluorescence quenching (Maksimov et al., 2016). As an example, the OCP-W288A mutant protein upon heterologous expression yields a mixture of species with a dominant fraction of photo-inactive red-purple protein, which is capable of PBS fluorescence quenching. At the same time, the absorbance spectra of many dark-adapted OCP species show spectral heterogeneity with admixtures of red states in the OCP^O^ form (Yaroshevich et al., 2021). However, mutational analyses occasionally revealed OCP variants with markedly reduced contamination by red states in the compact, dark-adapted OCP^O^ form. Such properties were observed for the OCP-Y201W mutant, whose spectrally pure, “super-orange” OCP^O^ state prove advantageous for femtosecond pump-probe UV-Vis spectroscopy to investigate the photocycle (Yaroshevich et al., 2021). For OCP-Y201W, the analysis of femtosecond transient UV-Vis absorption experiments combined with quantum chemical calculations led to the formulation of a reaction mechanism for the photochemistry in the early events that occur upon light-triggered H-bond breaking and involve the formation of an oxocarbenium ion on the ketocarotenoid (Yaroshevich et al., 2021).

Site-directed mutagenesis studies to probe the role of Trp288 and Tyr201 in OCP suffer from the limited ability to substitute aromatic amino acids within the standard amino acid repertoire. As a result, inaccurate conclusions have been drawn about which amino acid residues or their chemical properties are *necessary* for particular functional aspects, leaving unanswered the question of which chemical functionalities are *sufficient* for function. In this work, we performed a kind of “molecular surgery” in which a canonical amino acid residue was site-specifically replaced with a non-canonical analogue to remove the critical H-bond donor function of Trp288 of OCP with atomic precision. To this end, we used a recently evolved orthogonal pair (Tseng et al., 2020) consisting of a custom-tailored *Methanosarcina mazei* pyrrolysine tRNA synthetase (*Mm*PylRS) and its cognate tRNA to replace Trp288 in OCP with 3-benzothienyl-*L*-alanine (BTA), an isostructural analogue of tryptophan in which the imino nitrogen is substituted by a sulphur atom. The resulting OCP-W288_BTA protein is photoactive and, according to the X-ray crystal structure solved at 1.8 Å resolution, fully isomorphous to the wild-type protein, allowing us to parametrize the consequences of the absence of a single H-bond by spectroscopic, thermodynamic and computational methods.

## 2 Materials and methods

### 2.1 DNA constructs and molecular cloning

Engineering of the *Methanosarcina mazei* pyrrolysyl-tRNA synthetase (*Mm*PylRS) gene towards efficient loading of its cognate tRNA^Pyl^ with the non-canonical amino acid (ncAA) 3-benzothienyl-*L*-alanine (BTA) was carried out as described previously (Tseng et al., 2020). In detail, the *Mm*PylRS variant used here is denoted as *Mm*PylRS-13IYPER_SMG carrying the following set of mutations: T13I/T56P/H62Y/A100E/S193R/N346S/C348M/V401G conferring high BTA incorporation efficiency, and Y384F, which confers an increased aminoacylation rate of the aaRS enzyme, as known from previous studies (Ko et al., 2013). For further increase of aaRS processivity, the *Mm*PylRS cDNA sequence was codon-optimized for enhanced recombinant expression in *E. coli* [see (Tseng et al., 2020) for sequence information]. The cDNA of the *Mm*PylRS variant was cloned into plasmid pBU16 (ampicillin resistance) which contained pyrrolysyl–tRNA_CUA_ ( $t{RNA}_{CUA}^{Pyl}$ ) driven by a glnS’ promoter (Hauf et al., 2017).

The OCP gene (*slr 1963*) of *Synechocystis* sp. PCC 6803 was optimized for codon usage in *E. coli* by artificial gene synthesis (GeneOptimizer algorithm, GeneArt, Thermo Fisher Scientific, Waltham, MA, United States) and cloned into the first multiple cloning site of the pRSFDuet-1 vector (Novagen, Merck KgA, Darmstadt, Germany) by using *BamH*I and *Not*I restriction sites. The in-frame amber stop codon mutation Trp-288-TAG was generated using the Q5SDM Kit (New England Biolabs, Ipswich, MA, United States). For ease of eliminating truncated protein products, the 6xHis-Tag at the N-terminus was deleted and a new 6xHis-Tag was added to the C-terminus (resulting plasmid termed pRSFDuet-cTag herein, carrying kanamycin resistance) with an additional peptide linker sequence [the resulting C-terminal sequence was … NFAR**PGSS**HHHHHH* with the linker sequence PGSS in bold, as suggested by (de Carbon et al., 2015)] using the Q5SDM Kit (New England Biolabs, Ipswich, MA, United States). The resulting N-terminal sequence corresponds to the sequence of wild-type OCP from *Synechocystis* sp. PCC 6803 (amino acid sequence MPFTID … ). All cDNA constructs were verified by DNA sequencing (LGC genomics, Berlin, Germany). Synthesis of the carotenoid cofactor was accomplished using the p25crtO plasmid (carrying chloramphenicol resistance) which did not encode the *crtZ* gene but contained the contiguous gene cluster harboring the genes *crtY*, *crtI*, *crtB*, and *crtE* from *Pantoea ananatis* (formerly *Erwinia uredovora)* and the *crtO* gene from *Synechocystis* sp. PCC 6803 for echinenone/canthaxanthin synthesis (Misawa et al., 1995), and the strategy for expressing OCP variant proteins in echinenone/canthaxanthin-producing in *E. coli* strains followed the procedures described previously (Maksimov et al., 2016).

### 2.2 Protein expression and purification

To produce OCP with a site-specific ncAA incorporation, the three plasmids described above (pRSFDuet-cTag carrying the cDNA of the desired OCP variant, the p25crtO plasmid for ECN/CAN synthesis and the pBU16 plasmid carrying the components of the orthogonal translation system) were co-transformed into *E. coli* strain BL21 (DE3). Single colonies were picked and pre-cultured in 10 mL of Modified Tyler Medium (MTM: 2.5 g/L NH_4_Cl [46.7 mM], 0.71 g/L Na_2_SO_4_ [5 mM], 6.8 g/L KH_2_PO_4_ [50 mM], 7.1 g/L Na_2_HPO_4_ [50 mM], 6.0 g/L glycerol [65 mM], 2.5 g/L D-glucose [13.9 mM], 5 µM FeCl_2_, 2 µM CaCl_2_, 1 µM MnCl_2_, 1 µM ZnSO_4_, 0.2 µM CoCl_2_, 0.2 µM CuCl_2_, 0.2 µM NiCl_2_, 0.2 µM Na_2_MoO_4_, 0.2 µM Na_2_SeO_3_, 0.2 µM H_3_BO_3_, 0.2 µM vitamin B12, 0.2 µM nicotinic acid, 0.2 µM pyridoxine-HCl, 0.2 µM thiamine-HCl, 0.2 µM *p*-aminobenzoic acid, 5 nM folic acid, 5 nM riboflavin) (Tyler et al., 2005; Hoopes et al., 2015) supplemented with 100 µg/mL ampicillin (Amp), 50 µg/mL kanamycin (Kan) and 3734 µg/mL chloramphenicol (Cm) at 37°C in an orbital shaker at 200 rpm until OD_600_ reached 0.8–1.0. The pre-culture was transferred into 100 mL MTM supplemented with 100 µg/mL Amp, 50 µg/mL Kan, and 3734 µg/mL Cm and grown at 37°C in an orbital shaker at 200 rpm until OD_600_ reached 0.6–0.8. Then, 2 mM of the ncAA was added to the culture, which subsequently was further incubated at 37°C for 30 min. Subsequently, target gene expression was induced by adding 1 mM isopropyl-β-*D*-thiogalactopyranoside (IPTG) and incubation at 25°C and 250 rpm proceeded for 72 h. Cells were harvested by centrifugation at 10,000 × g at 4°C for 20 min and frozen until use. For the expression of the OCP-W288A variant, the same conditions were used except the steps of non-canonical amino acid-related biochemistry.

The frozen cell pellets were resuspended in phosphate buffered saline (1xPBS: 137 mM NaCl, 2.7 KCl, 12 mM phosphate at pH 7.4) and supplemented with 0.5 mg/mL lysozyme (Ovobest, Neuenkirchen-Vörden, Germany) and protease inhibitor solution (1 mM benzamidine, 1 mM ε-amino-caproic acid). Cell lysis was performed by an Omniruptor 4,000 sonifier (OMNI International, Kennesaw, GA, United States) with 5 cycles of 3 min sonification and 3 min cooling breaks on ice between each cycle, the tip power was tuned to 80%. To remove the cell debris, the cell solution was centrifuged at 18,000 × g at 4°C and the clarified supernatant was loaded on a 5 mL Co^2+^-HiTrap Talon crude column (GE Healthcare, Chicago, IL, United States) by using a peristaltic pump. The protein was eluted by an imidazole-containing phosphate buffer (1xPBS, 350 mM imidazole pH 7.4). To remove carotenoid-free apoprotein and other contaminants, the protein solution was purified by hydrophobic interaction chromatography (HIC) by using a Hiprep 16/10 Phenyl HP column (GE Healthcare) as follows: The protein solution was dialyzed overnight in HIC buffer A (0.5 M (NH_4_)_2_SO_4_, 0.1 M urea, 5 mM phosphate pH 7.5), loaded on the HIC buffer A-equilibrated column and finally eluted by a binary gradient of HIC buffer B (0.1 M urea, 5 mM phosphate pH 7.5). Finally, the collected and concentrated protein solution was purified using a Superdex 200 Increase 10/300 column (GE Healthcare) to separate the monomeric orange from the dimeric pink protein species.

### 2.3 Mass spectrometry

To identify ncAA incorporation into OCP by *Mm*PylRS, intact protein masses were analyzed by an electrospray ionization mass spectrometry (ESI-MS) source (Waters H-class instrument). The proteins were eluted through an Acquity UPLC protein BEH C4 column (300 Å, 1.7 μm, 2.1 mm × 50 mm) with a flow rate of 0.3 mL/min. The resulting ions were analyzed by a Waters XEVO G2-XS QTof analyzer (Waters Corp., Milford, MA, United States). Spectra deconvolution was performed using MaxEnt 1 employing the maximum entropy deconvolution algorithm. Observed protein masses were compared to theoretical values determined by the Expasy ProtParam tool (Gasteiger et al., 2005).

### 2.4 Carotenoid extraction and HPLC analysis

For analysis of the carotenoid content of OCP holoprotein variants, 50 µL of concentrated protein samples were mixed with 1 mL acetone and centrifuged at maximum speed at 4°C to spin down precipitated protein. The yellowish supernatant was evaporated in a centrifugal vacuum concentrator (Eppendorf, Hamburg, Germany) until all acetone was removed and the carotenoid sample was precipitated as red crystals. The remaining water solution was removed and the carotenoid crystals were re-dissolved in 50 µL acetone. The carotenoid-rich solution was transferred into a vial for HPLC analysis. HPLC was performed on an UFLC NexeraX2 system (Shimadzu, Kyoto, Japan) by using a Accucore™ C30 column (Thermo Fisher Scientific, 250 mm × 2.1 mm, 2.6 µm particle size, pore size 150 Å). As mobile phase two eluents A (methanol:water, 95:5) and B (methanol:THF, 7:3) were used with the following protocol to elute carotenoids: 0—4.3 min 0% buffer B, 4.3—8.6 min 0→100% buffer B, 8.6—15.6 min 100% buffer B, 15.6—20.1 min 0% buffer B and a flow rate set to 0.4 mL/min. The eluted carotenoids were verified by mass spectroscopy to correlate elution times to specific carotenoid species, and by thin-layer chromatography by comparison with reference samples.

### 2.5 UV/VIS spectroscopy and kinetic analysis

Absorption spectra were recorded using a Maya2000Pro spectrometer (Ocean Insight, Orlando, FL, United States) coupled by a fiber to a deuterium tungsten light source (Sarspec, Vila Nova de Gaia, Portugal) and a cuvette holder (CVH100, Thorlabs, Bergkirchen, Germany). For kinetic measurements, a temperature-controlled cuvette holder qpod2e (Quantum Northwest, Liberty Lake, WA, United States) was fiber-coupled to a CCS100/M spectrometer (Thorlabs) and a SLS201 L/M tungsten light source (Thorlabs). For illumination with actinic light, a 3W (Avonec, Wesel, Germany) light-emitting diode (LED) with a maximum emission at 455 nm was used. Analysis of the observed time constants for photoconversion (OCP^O^→OCP^R^) and back-relaxation (OCP^R^→OCP^O^) was performed based on a simple two-state kinetic model described in (Maksimov et al., 2015) as follows:

$${OCP}^{O}\begin{array}{c} F∙\sigma \\ \;⇌\;\\ r \end{array}{OCP}^{R}$$ (1)

In a two-state model with first order rate constants, the total (observed) relaxation rate *k*_*obs*_ during photoconversion by light is the sum of the light-dependent forward rate constant (the product of illumination intensity (fluence) *F* and the photoconversion cross-section *σ*) and the light-independent relaxation rate *r*. Thus, whereas the rate constant of the light-independent back-relaxation can be directly measured, the rate-constant of the light-dependent forward reaction ( F∙σ ) is calculated as:

$$F∙\sigma =k_{obs}-r$$ (2)

As a prerequisite, the photoswitching reaction always needs to be followed at the same illumination intensity at the sample, which was obeyed in the present experiments.

### 2.6 Resonance Raman (RR) spectroscopy

Spectra were recorded at −140°C (Linkam cryostat, Resultec, Illerkirchberg, Germany) using a Fourier-Transform Raman spectrometer RFS-100/S (Bruker, Rosenheim, Germany) equipped with a 1,064 nm cw NdYAG laser (Compass 1064-1500N, Coherent LaserSystems, Santa Clara, CA, United States) as described (Moldenhauer et al., 2017). Although 1,064 nm excitation is not in resonance with chromophore absorption, the chromophore signals are ∼1:1,000 enhanced over the protein bands justifying use of the term “resonance Raman” (RR) spectroscopy for our pre-resonant conditions. For each RR spectrum, 1,000 single scans were averaged. The samples were prepared at a concentration of 230 µM (wild-type OCP) and 243 µM (OCP-W288_BTA) in 1xPBS, pH 7.4. For photoconversion, a 3-W LED (Avonec, Wesel, Germany) was used.

### 2.7 Crystallization and X-ray data collection

Protein crystallization was carried out as sitting drop experiments by mixing 300 nL of protein and well solution each. A thawed aliquot of OCP-W288_BTA with a concentration of 2 mg/mL stored in 1xPBS Buffer (137 mM NaCl, 2.7 KCl, 12 mM phosphate, pH 7.4) was used for crystallization. The crystal used for structure determination grew in the presence of 0.2 M MgCl_2_, 0.1 M Tris pH 7.0% and 10% (w/v) PEG 8000. Crystals were mounted on micro mounts and flash-frozen in liquid nitrogen before transport. The synchrotron X-ray diffraction data was collected at beamline P14 operated by EMBL Hamburg at the PETRA III storage ring (DESY, Hamburg, Germany).

### 2.8 Crystal structure solution and refinement

Diffraction data were processed using X-ray Detector Software (XDS) (Kabsch, 1993). Subsequent data reduction was performed using the CCP4i2 pipeline running AIMLESS (Evans and Murshudov, 2013) and PHASER (McCoy et al., 2007) for molecular replacement using the OCP wild-type structure (PDB: 5TUW) as search model. Afterwards, the structure was refined by several rounds of manual model building and optimization using Coot (Emsley and Cowtan, 2004) followed by refinement and quality check with phenix.refine (Afonine et al., 2012). The refinement strategy involved rigid body refinement, individual B-factors, TLS parameters and occupancies. OCP-W288_BTA coordinates and structure factors were deposited in the RCSB protein database under the PDB accession code 7ZXV. The structure of the BTA side chain was obtained by quantum chemical (QC) calculations and the corresponding coordinates were used in structure refinement. Crystal structure depictions were produced using PyMOL 2.5.2.

### 2.9 Quantum chemical (QC) calculations

To obtain the equilibrium geometry for BTA, QC calculations with ωB97x (Mardirossian and Head-Gordon, 2014)/6–311G** (Ditchfield et al., 1971) theory level were used. Calculations were done using the ORCA 4.2 software package (Neese et al., 2020).

The calculation of the molecular complex between BTA, tryptophan and the xanthophyll was performed with quantum dynamics (QD). Two systems consisting of tryptophan/BTA, tyrosine, and the xanthophyll were taken from the PDB crystal structures of 3MG1 and 7ZXV, respectively. Both complexes were optimized with constraints applied to nine atomic nuclei: three of the heavy atoms for both amino acid backbones, and three methyl groups at the C1 and C5’ atoms of ECN. The optimized systems were passed for conducting 10 ps QD calculations with integration time step of 1 fs using the ωB97x/def2-SVP (Weigend and Ahlrichs, 2005) method. Simulations were performed at 300 K and 1 bar pressure using the Bussi-Parrinello Langevin dynamics (Bussi and Parrinello, 2007). Calculation was done with TeraChem v1.9 (Seritan et al., 2020) software using the super-computing center at Lomonosov Moscow State University.

## 3 Results and discussion

**FIGURE 2:**
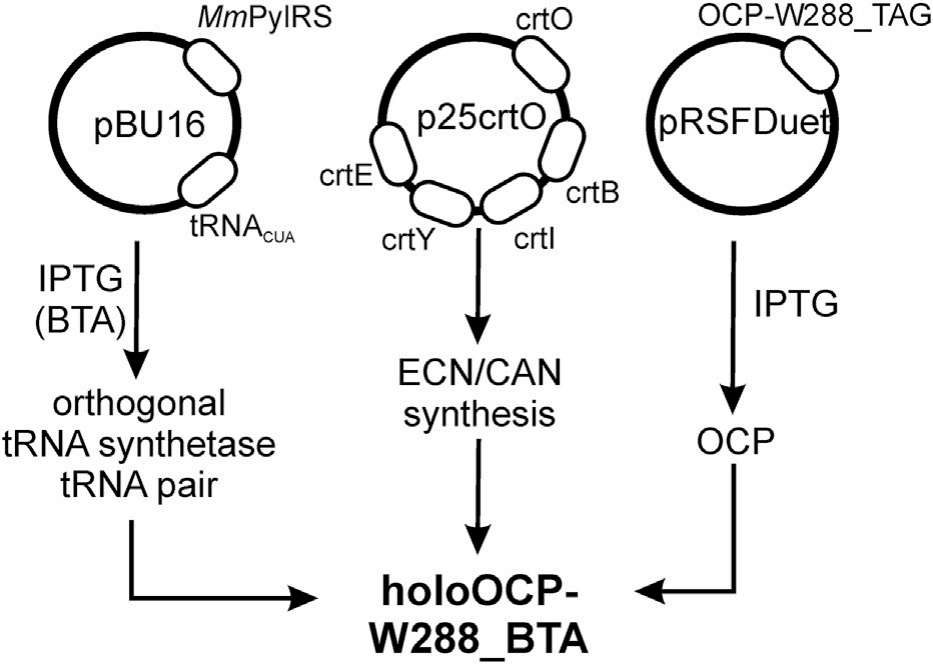
Schematic of heterologous expression of OCP-W288_BTA by stop codon suppression in *E. coli*. Heterologous expression was performed based on an orthogonal pair consisting of a specifically tailored *Mm*PylRS and its cognate tRNA^Pyl^, which incorporates the non-canonical amino acid 3-benzothienyl-*L*-alanine (BTA) with high efficiency. The *Mm*PylRS gene and the cognate tRNA^Pyl^ sequence are in the pBU16 plasmid (carrying ampicillin resistance). Plasmid p25crtO harbors a contiguous gene cluster consisting of the genes *crtY, crtI, crtB*, and *crtE* from *Pantoea ananatis* (formerly *Erwinia uredovora*) and the *crtO* gene from *Synechocystis* sp. PCC 6803 for echinenone/canthaxanthin synthesis (chloramphenicol resistance). The pRSFDuet-cTag plasmid (kanamycin resistance) carried the cDNA of the OCP construct. These plasmids were co-transformed into *E. coli* strain BL21 (DE3).

**FIGURE 3:**
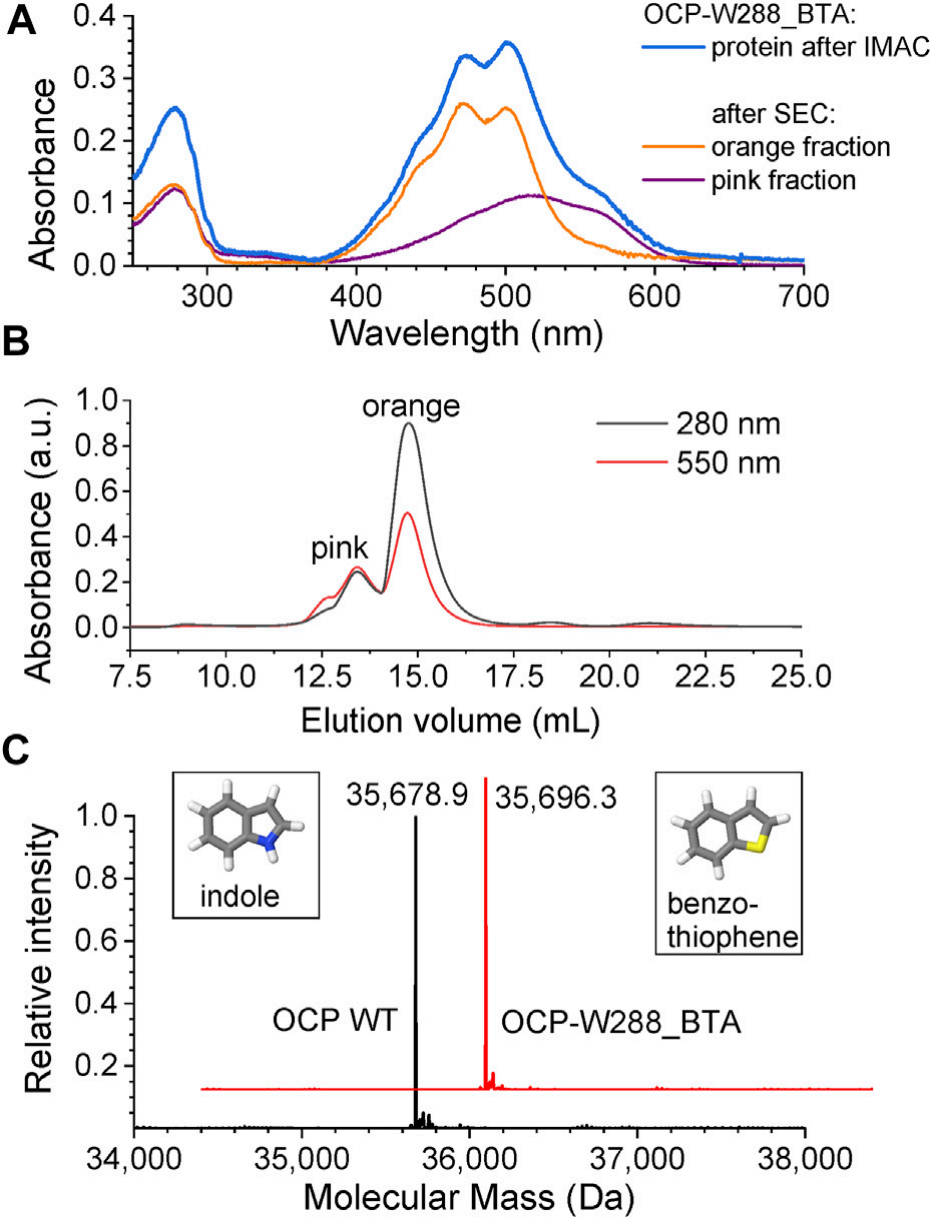
Chromatographic separation and mass spectrometric analysis of OCP-W288_BTA protein. **(A)** Absorbance spectra of the OCP-W288_BTA sample after His-tag affinity elution (immobilized metal affinity chromatography, IMAC) (blue) and the orange [OCP-W288_BTA(O)] and pink [OCP-W288_BTA(P)] species after SEC. **(B)** The orange and pink OCP-W288_BTA samples obtained by SEC. The elution profiles were observed at two different wavelengths as indicated. **(C)** Deconvoluted ESI-MS profiles of full-length OCP (wild-type) and OCP-W288_BTA (orange species). The observed molecular masses were: Wild-type OCP (35,678.9 Da, OCP-W288_BTA 35,696.3 Da. The calculated masses (-Met) were 35,679.8 Da and 35,696.0 Da, respectively. The structures of indole and benzothiophene, the side chains of Trp and BTA, respectively, are shown in the insets.

We used an *Escherichia coli* expression host for the biosynthesis of the BTA-containing OCP holoprotein variant (OCP-W288_BTA). Our expression system is based on three plasmids (Figure 2). Affinity chromatography using His-tag yielded an intensely stained protein solution (blue spectrum in Figure 3A) showing characteristic features of dark-adapted OCP with a small amount of a red-shifted species, which shows up as a shoulder at 570 nm (Sluchanko et al., 2017a). After an additional purification step with preparative size-exclusion chromatography (SEC), an orange and a pink protein species could be separated based on their different elution profiles (Figure 3B) (Sluchanko et al., 2017b). While the pink species [OCP-W288_BTA(P)] eluted earlier, the orange fraction [OCP-W288_BTA(O)] corresponded to the retention time of an OCP monomer. Figure 3A shows the representative spectra of a protein preparation that contained approximately 35% pink and 65% orange species. The purified proteins of the orange OCP-W288_BTA(O) variant and wild-type OCP were subjected to electrospray LC-MS analysis (Figure 3C), which revealed 100% incorporation of BTA in the variant protein.

The carotenoid content of the protein samples was determined by HPLC (Table 1). While wild-type OCP almost exclusively contained ECN, the orange OCP-W288_BTA(O) species had two peaks of approximately the same amplitude (54%/46%) corresponding to ECN and CAN. Different amounts of ECN/CAN in OCP samples obtained from xanthophyll-producing *E. coli* strains is a common phenomenon (de Carbon et al., 2015) and may depend on the employed β-carotene ketolase gene or on the expression level of OCP in the cells. However, the photoswitching ability is not affected by the presence of ECN or CAN (de Carbon et al., 2015) (see below). In contrast, the pink OCP-W288_BTA(P) species contained more than 96% CAN.

**TABLE 1:** Carotenoid content determined by HPLC (see chromatograms in Supplementary Figure S1).

Sample	Carotenoid	Retention time (min)	Relative content (mean ± S.D) in %
OCP wild-type	canthaxanthin	7.6	3.0 ± 3.1
	**echinenone**	**9.7**	**91.7 ± 2.5**
	*ECN prepeak* ^[^ ^a^ ^]^	9.5	5.3 ± 1.7
OCP-W288_BTA orange	**canthaxanthin**	**7.5**	**53.9 ± 4.8**
	**echinenone**	**9.7**	**42.4 ± 4.0**
	*ECN prepeak* ^[^ ^a^ ^]^	9.5	3.6 ± 0.8
OCP-W288_BTA pink	**canthaxanthin**	**7.6**	**90.9 ± 6.8**
	echinenone	9.7	8.5 ± 7.3

^a^ The *ECN, prepeak* species was not further characterized. It may consist of c*is*-echinenone, which may have formed during the extraction procedure, the relative content depends on parameters such as sample concentration or residual water content—it is not considered to be present as *cis*-echinenone in the protein. Bold values denote the dominant carotenoid species.

**FIGURE 4:**
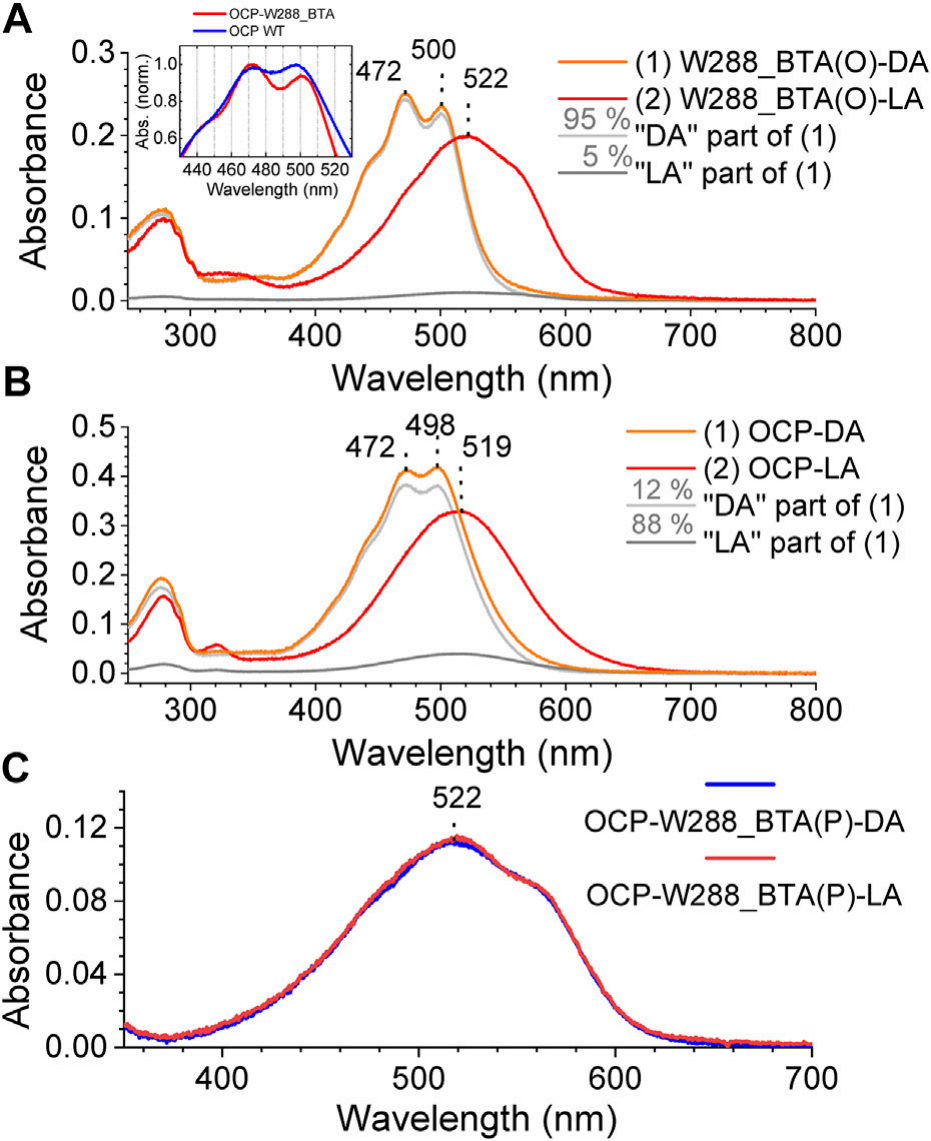
Photoswitching of the OCP-W288_BTA(O) and OCP-W288_BTA(P) species. **(A)** Absorption spectra of the OCP-W288_BTA(O) fraction of the protein in the dark-adapted (DA) and light-adapted (LA) states (orange and red curves, respectively) and decomposition of the DA spectrum into 95% orange (light grey) and 5% red (dark grey) spectral components. **(B)** Absorbance spectra of wild-type OCP in the dark-adapted (DA) and light-adapted (LA) states (orange and red curves, respectively) and decomposition of the DA spectrum into 88% orange (light grey) and 12% red (dark grey) spectral components. The inset in **(A)** shows the spectra of OCP wild-type and OCP-W288_BTA(O) at higher magnification. **(C)** Absorption spectra of the pink fraction of OCP-W288_BTA protein in the dark-adapted (DA) state (magenta) and after 5 min illumination (LA) with blue light (violet).

When both the orange and pink OCP-W288_BTA protein fractions were illuminated with blue light (3-W LED, 455 nm) at 15°C, the OCP-W288_BTA(O) species was photoswitchable (Figure 4A) like wild-type OCP (Figure 4B), whereas no spectral changes were observed for the OCP-W288_BTA(P) species (Figure 4C). Spectral decomposition showed that the spectrum of the dark-adapted OCP-W288_BTA(O) species contained a much smaller contribution of a red non-photoswitchable species compared with the wild-type OCP (Figure 4A) than the dark-adapted wild-type OCP (5% vs. 12%, respectively) (Figure 4B). The absorption spectrum of the orange OCP-W288_BTA(O) species exhibits a distinct vibrational fine structure, with even more pronounced vibrational bands than the wild-type OCP^O^ (Figure 4A, inset). These features of the OCP-W288_BTA(O) protein, a spectrally more homogenous dark-adapted state and a deeper vibrational substructure are typical attributes of so-called “super-orange” OCP species, as first described for the OCP-Y201W mutant (Yaroshevich et al., 2021).

In the OCP-Y201W mutant protein, the lack of one H-bond donor [according to the X-ray crystal structure solved in (Yaroshevich et al., 2021), the introduced Trp residue is too far away from the C=O group of the ketocarotenoid] leads to an increase in the importance of the remaining H-bond (from the C=O group of the carotenoid to the N-H group of Trp288), making the ground state less susceptible to spontaneous carotenoid fluctuations. In the OCP-W288_BTA variant, the same effect could also occur in the opposite direction: Removal of the imino nitrogen by a sulphur atom also strengthens the importance of the remaining H-bond to Tyr201.

The spectra of the OCP-W288_BTA(P) species and the light-adapted OCP-W288_BTA(O) species are very similar (Supplementary Material S1, Supplementary Figure S2). This suggests that the spectroscopic properties of the OCP-W288_BTA(P) species are determined by features attributable to domain separation and complete translocation of the chromophore into the NTD, as present in the photoactivated form of OCP. The aforementioned spectra are also very similar to the absorbance spectrum of the Red Carotenoid Protein when it coordinates CAN (i.e., the OCP-NTD holoprotein produced by heterologous expression in *E. coli*) (Moldenhauer et al., 2017), but slightly blue-shifted and distinct from the absorbance spectrum of the pink fraction of the OCP-W288A mutant protein previously characterized (Maksimov et al., 2016) (Supplementary Material S1, Supplementary Figure S2).

**FIGURE 5:**
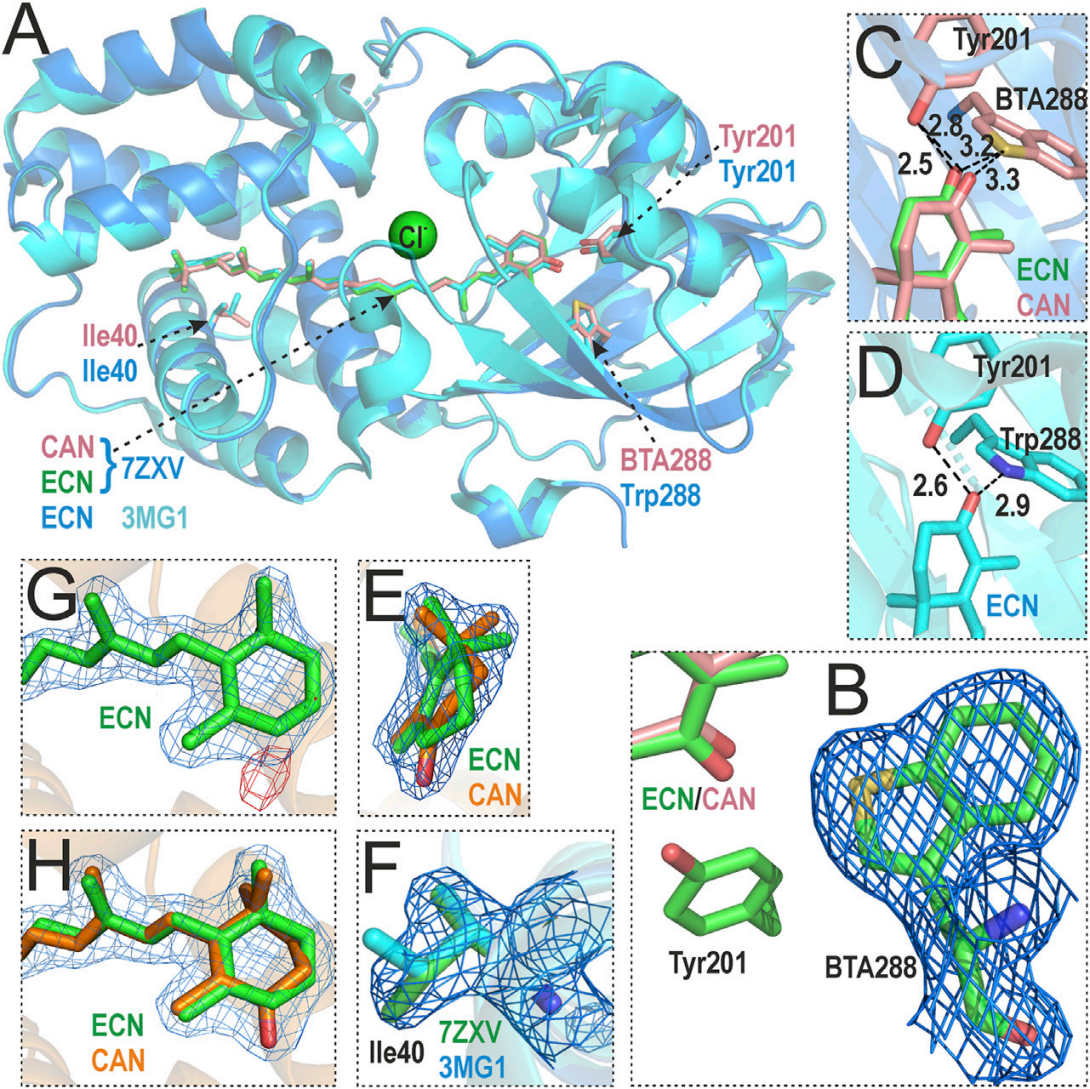
Crystal structure of the OCP-W288_BTA variant (PDB: 7ZXV) and comparison with wild-type *Synechocystis* OCP (PDB: 3MG1). **(A)** Overlay of OCP-W288_BTA (blue) and wild-type OCP (cyan) shows no global structural changes. Residues Tyr201, Trp/BTA288 and Ile40 are shown as ball-and-stick models (note the different colors of the C atoms: rose/orange/green in 7ZXV, and cyan in 3MG1). A Cl^−^ ion discovered in the 7ZXV structure is shown in green. **(B)** Sigma-A-weighted 2*mFo*-*DFc* electron density map around BTA288, reflecting the distinct ring geometry of the BTA side chain. **(C,D)** Cofactor coordination in detail for OCP-W288_BTA **(C)** and wild-type OCP **(D)**. The distance between BTA288 and the xanthophyllic cofactors increases only slightly from 2.9 Å to 3.2/3.3 Å compared to coordination by Trp288 in wild-type OCP, the distance between Tyr201 and ECN is comparable to wild-type OCP and slightly increased for CAN. **(E)** The 2*mFo-DFc* electron density map is shown around the ECN/CAN end rings in the NTD to illustrate the different ring geometries corresponding to the *sp* ^2^/*sp* ^3^ hybridization state of the carbonyl carbon atom in CAN/ECN. **(F)** The 2*mFo-DFc* electron density map around Ile40 in 7ZXV shows a different rotamer state of the side chain (green) than in the 3MG1 structure (cyan). **(G)** The 2*mFo-DFc* (blue) and *Fo-Fc* (red) electron density maps around the xanthophyll cofactor in OCP-W288_BTA, in which only ECN is fitted, clearly show the difference electron density attributable to a missing keto oxygen atom (red). **(H)** The 2*mFo-DFc* electron density map in which both ECN and CAN were fitted showed occupancies of 45% and 55%, respectively, after refinement.

We were able to crystallize the orange species of OCP-W288_BTA containing a mixture of ECN and CAN and determined its structure (PDB: 7ZXV) with a resolution of 1.8 Å (see Table 2 for crystallographic information) by molecular substitution with the structure of wild-type OCP as a template (PDB: 5TUW) containing an ECN cofactor. As expected, the overall structure is very similar to the wild-type OCP, with RMSD values of 0.180 Å and 0.203 Å for monomer and dimer alignments at the Cα-positions, respectively. A close inspection of Figure 5A clearly shows that the BTA mutation has no significant effect on the global OCP structure, and the different geometry of the BTA side chain (Figure 5B; C-S-C angle of 92.5°) compared to Trp288 (C-N-C angle of 109°) is nicely reflected by the electron density (Figure 5B and Supplementary Material S1, Supplementary Figure S3). The introduced BTA residue matches well with its native Trp288 counterpart, resulting in an almost identical spatial arrangement (i.e., isomorphic replacement). Interestingly, the distance to the keto oxygen atoms of the ECN/CAN cofactors increases only slightly from 2.9 Å in the wild-type protein to 3.2 Å and 3.3 Å, respectively (Figures 5C, D), despite the potential repulsion of the lone electron pairs of sulphur, which, however, do not appear as a significant electron density in the *2mFo-DFc* map (Figure 5B, and Supplementary Material S1, Supplementary Figure S3). In addition, the distance between Tyr201 and the keto oxygen of ECN and is almost identical at 2.5 Å compared to 2.6 Å in wild-type OCP. However, the distance to CAN is slightly increased to 2.8 Å (Figures 5C, D). Here, the carbonyl carbon atom of CAN has an *sp* ^*2*^ hybridization state, whereas the corresponding carbon atom of ECN is *sp* ^*3*^-hybridized, which affects the overall ring geometry in the NTD (Figure 5E) and is probably responsible for the small shift at the distal end of the xanthophyll in the CTD. Nevertheless, the protein environment of the xanthophylls seems to be unaffected by the presence of an additional keto function in the NTD, and the CAN configuration is almost identical to the one of the CAN cofactor in another *Synechocystis* wild-type OCP structure (PDB: 4XB5; Supplementary Material S1, Supplementary Figure S4). The only observable difference is that Cδ of Ile40 seems to be rotated 120° compared to the wild-type (Figure 5F), yet this does not result in a subsequent conformational change. Overall, the differences due to the BTA mutation and the different cofactor notably result in very minor structural changes.

**TABLE 2:** OCP-W288_BTA X-ray data collection and refinement statistics.

Data collection	
PDB ID	7ZXV^1^
Diffraction source	P14 beamline (EMBL Hamburg, Germany) at Petra III storage ring (DESY, Hamburg, Germany)
Wavelength (Å)	0.9763
Space group	P 3_2_ 2 1
Cell dimensions: a, b, c (Å)	83.195, 83.195, 88.648
α, β, γ (°)	90.00, 90.00, 120.00
Resolution range (Å) *	72.05-1.80 (1.84-1.80)
Unique reflections	32,925 (1940)
R_merge_	0.114 (4.998)
R_pim_	0.037 (1.628)
R_meas_	0.120 (5.574)
<I/σ>	13.7 (0.6)
CC_1/2_	0.999 (0.477)
Wilson B factor (Å^2^)	37.07
Completeness for range (%)	98.7 (100)
Redundancy	20.2 (20.1)
Refinement	
Resolution range (Å)	55.91-1.80 [55.91-4.12] (1.85-1.80)
No. of reflections: total	32,864 [2,789] (2,587)
“working” set	31,224 [2,627] (2,458)
“free” set	1,640 [162] (129)
R_work_ (%)	17.30 [13.89] (44.76)
R_free_ (%)	22.23 [18.94] (52.70)
Mean B value (overall Å^2^)	40.75
Completeness (working + test) (%)	98.58
No. of non-H atoms: protein/ligands/solvent	2,464/83/308
R.m.s.d. bond lengths (Å)/angles (°)	0.012/1.079
Ramachandran favored/outliers (%)	99.03/0.0
Molprobity/All-atom clashscore	1.156/2.3

* *Statistics* for the lowest and highest resolution shells are indicated in square brackets and parentheses, respectively.

During refinement, the electron density of a Cl^−^ ion was delineated that was also present in another OCP mutant (Y201W, PDB: 6T6K) at the same position; which, however, is not present in the 3MG1 structure. The only potential OCP interaction partner in the proximity is Thr275. Interpretation of the xanthophyll cofactor electron density based on the wild-type structure with only one ECN molecule resulted in a difference density in the NTD (Figure 5G). Subsequently, the electron density was interpreted with an additional CAN molecule and occupancy refinement (Figure 5H), which led to the conclusion that both xanthophylls are present in the OCP-W288_BTA mutant: The ratio of ECN/CAN ratio (45:55) corresponds to the ratio of xanthophylls extracted from the purified protein (Table 1).

To gain insight into the character of the interaction in the ECN-Tyr201-BTA288 complex, we performed a 10 ps quantum dynamics (QD) calculation. A comparison of the distances between the key atoms in the ECN-Tyr201-Trp288/BTA complexes (Supplementary Material S1, Supplementary Figure S5) clearly shows the absence of the energetically favourable interaction between the sulphur atom in BTA and the keto oxygen in ECN. The distance of 3.3 Å between the keto oxygen and the sulphur atom indicates the dispersion nature of the interaction in the ECN-BTA complex. Despite the high polarizability of the bulky sulphur atom, such an interaction does not significantly affect the OCP-W288_BTA absorption spectrum in the dark-adapted state. Moreover, a slight trend toward shortening of the ECN-Tyr201 H-bond is observed in the presence of BTA. This effect is consistent with the hypothesis that the absence of a competing H-bond donor for the keto oxygen enhances the interaction with the remaining H-bond donor (Yaroshevich et al., 2021). In the case of OCP-W288_BTA, the remaining H-bond is formed with Tyr201, making it similar to the previously reported OCP-Y201W mutant (Yaroshevich et al., 2021).

With the introduction of BTA into OCP, several new effects could be expected from the appearance of a bivalent sulphur atom in close proximity to the keto group of the bound xanthophyll. For example, the electrostatic interaction between the sulphur atom of a methionine and the carboxylate oxygen atom was suggested to play a major role in enzymatic catalysis (Taylor and Markham, 1999). Further analysis of structural data revealed that the bivalent sulphur atom forms specific non-bonded (sometimes called hypervalent) interactions with non-hydrogen atoms (O, N, C) within the protein backbone (Iwaoka and Isozumi, 2012). These relatively weak interactions can be classified into several types: Two-center-three-electron (Mishra et al., 2009), bifurcated (Zhang and Wang, 2009) and sulphur-carbene (Zhao et al., 2011) interactions. In addition, a theoretical analysis has shown that electron deficient, bivalent sulphur atoms can interact with electron donors by accepting their lone pair into low-lying σ* orbitals (Beno et al., 2015). All these types of specific interactions are sensitive to the mutual orientations of the interacting counterparts and expected to be energetically significant at S···O distances less than 3 Å (Iwaoka and Isozumi, 2012; Beno et al., 2015). However, with respect to the interaction of low-lying σ* orbitals of the C–S in the BTA bond with electron donors, we note that, first, the electron deficiency at the sulphur atom in BTA should be low [see Figure 8 in (Beno et al., 2015)], and, second, the sulphur (acceptor), oxygen (donor), and carbon (covalently bound to the sulphur in BTA) should be collinear (180° C-S-O angle) for a favourable interaction to occur. Yet, the corresponding C-S-O angle from the 7ZXV crystal structure deviates substantially (160°), arguing against a significant contribution of the aforementioned non-bonded interactions.

**FIGURE 6:**
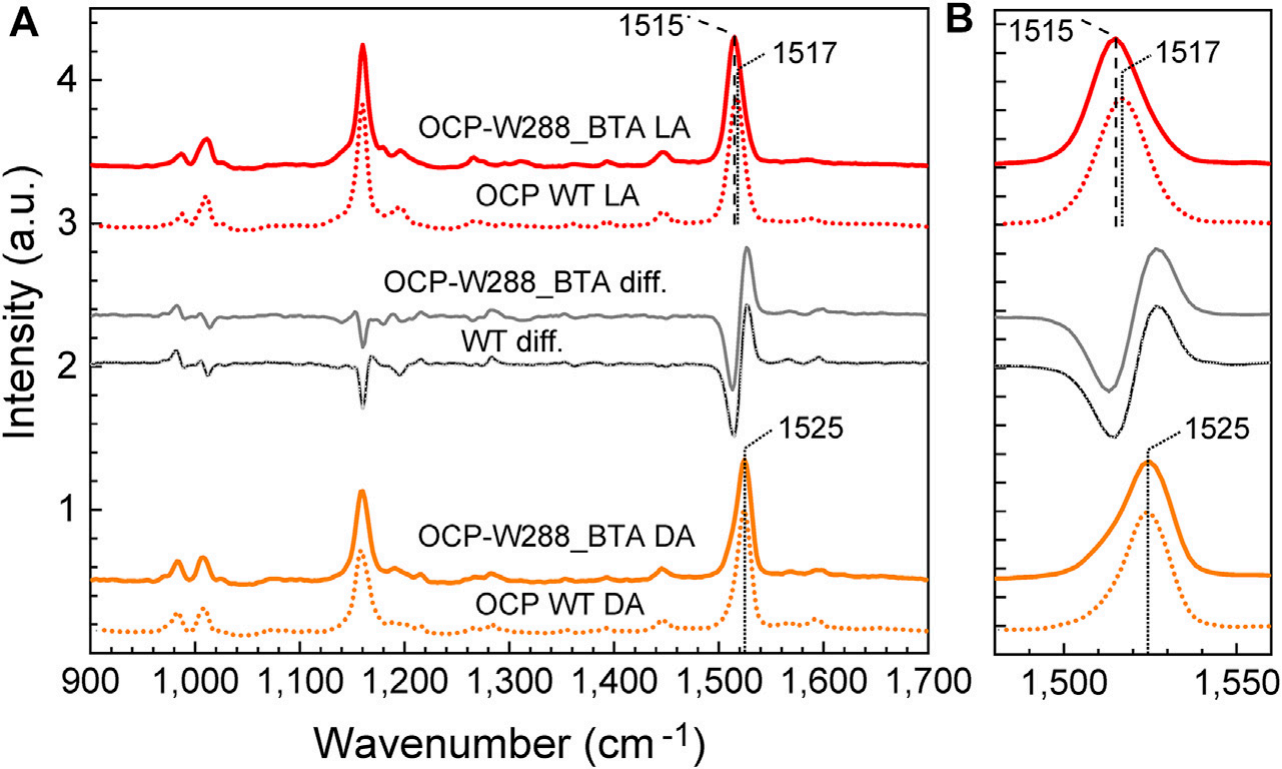
Resonance Raman spectra of wild-type OCP and the OCP-W288_BTA variant in the dark-adapted and illuminated states. **(A)** Resonance Raman spectra of wild-type OCP (dotted curves) and the OCP-W288_BTA variant (solid curves) in the dark-adapted (DA, orange curves) and light-adapted (LA, red curves) states. The peak wavenumbers of the ν_1_ band are indicated. The grey curves show the difference spectra DA minus LA. The region of the ν_1_-band is shown in **(B)** at higher magnification. All dark- and light-matched spectra were intensity normalized based on the amplitude of the ν_1_-band and vertically offset for clarity.

Resonance Raman spectra of the photoswitchable OCP-W288_BTA protein were recorded for dark- and light-adapted samples and compared with those of wild-type OCP (Figure 6), and those in the literature (Leverenz et al., 2014; Kish et al., 2015; Maksimov et al., 2016). In agreement with the established band assignment for carotenoids (Schlücker et al., 2003), the ν_1_ band position around 1,525 cm^−1^ is mainly due to C=C double bond stretching vibrations of the conjugated polyene chain, while the ν_2_ band around 1,160–1,180 cm^−1^ consists of contributions from stretching vibrations of C–C single bonds coupled with C–H in-plane bending modes. The ν_3_ band at 1,000 cm^−1^ arises from in-plane rocking vibrations of methyl groups on the conjugated polyene chain, coupled with in-plane bending modes of the neighboring C–H groups, and collectively considered as a fingerprint of the conjugated end-cycle configuration. Finally, the ν_4_ band around 960 cm^−1^ stems from C–H out-of-plane wagging motions coupled with C=C torsional modes (out-of-plane twists of the carbon backbone). When the conjugated system of the carotenoid is planar, these out-of-plane modes are not coupled with the electronic transition, and these bands are not resonance-enhanced. However, distortions around C–C single bonds increase the coupling of these modes with the electronic transition, leading to an increase in their intensity (Kish et al., 2015). The ν_1_, ν_2_, ν_3_ and ν_4_ bands of dark-adapted wild-type OCP and the OCP-W288_BTA variant were at the same positions and had very similar amplitudes (Figure 6).

After photoconversion, there was a clear red shift of the ν_1_ band from 1,525 cm^−1^ to 1,517 cm^−1^ for wild-type OCP, and even to 1,515 cm^−1^ for OCP-W288_BTA, which is consistent with the correlation between the position of the ν_1_ band and the position of the maximum of the chromophore’s absorbance spectrum (Kish et al., 2015). Theoretical studies have shown that this correlation is due to a combination of two effects: (i) the rotation of the β-ionone rings with respect to the conjugated system (Mori, 2016) and (ii) the local electric field in the chromophore-binding pocket (Gamiz-Hernandez et al., 2015). The red form of OCP-W288_BTA even appears to be slightly more red-shifted than wild-type OCP, which can be attributed to the effect of CAN embedment, which also causes a slight red-shift in the absorbance spectrum compared to ECN embedment (de Carbon et al., 2015). After photoconversion, both proteins show a decrease in the intensity of the ν_4_ band since the carotenoid assumes a more planar configuration in the photoactivated state. The dark-minus-light difference spectra for the wild-type OCP and the BTA-bearing variant are virtually identical, leaving only the slight red shift in the ν_1_ band of the photoactivated OCP-W288_BTA by 2 cm^−1^ as a difference (Figure 6B).

**FIGURE 7:**
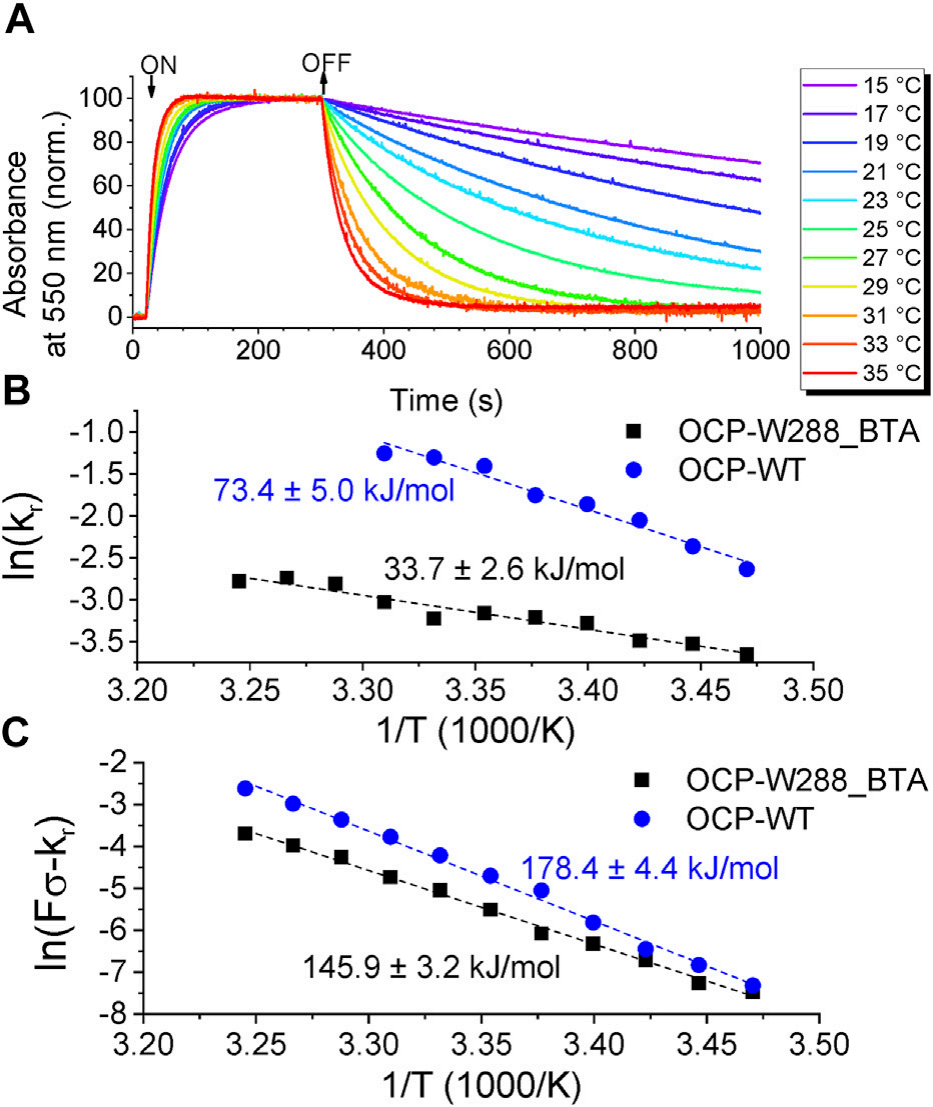
Photoconversion of OCP-W288_BTA. **(A)** Normalized time-courses of the absorption changes at 550 nm at temperatures from 15°C to 35°C upon OCP^O^→OCP^R^ photoconversion and subsequent relaxation from OCP^R^ to OCP^O^. **(B)** Arrhenius plot of the natural logarithm of the rate constants of OD_550_ changes ( F·σ ) minus the rate constant of recovery at the same temperature ( r ) during OCP^O^-to-OCP^R^ photoconversion (obtained with the same illumination intensity) against the reciprocal temperature. **(C)** Arrhenius plot of the natural logarithm of the rate constants ( r ) of OD_550_ changes during relaxation from the steady state reached after constant illumination back to the dark-adapted state against the reciprocal temperature. The line fits to the data sets are included yielding the activation energies indicated in the figure panels.

Next, we investigated the kinetics of transitions between the dark-adapted OCP^O^ state and the light-adapted OCP^R^ state and monitored the OCP^O^→OCP^R^ photoconversion and subsequent recovery as the change in absorbance at 550 nm, always at the same illumination intensity (fluence *F*) at different temperatures (Figure 7A). Fitting the rising and decaying phase of the curves with a monoexponential function yielded the rate constants for photoconversion and recovery [rate constants *F∙σ* and *k*_*r*_, respectively, see the procedure described by (Shirshin et al., 2017)]. The resulting Arrhenius diagrams for the photoconversion and recovery rate constants for OCP-W288_BTA variant and wild-type OCP are shown in Figures 7B, C, respectively. The data show that the OCP^O^→OCP^R^ photoconversion of OCP-W288_BTA is significantly slowed compared with wild-type OCP. Moreover, the activation energy (E_A_) for OCP-W288_BTA determined from the Arrhenius plots is substantially reduced by ∼40 kJ/mol compared with wild-type OCP. The same trend is observed for the rate constant for recovery to the ground state: The rate constant of OCP-W288_BTA is slowed down compared to wild-type OCP, and E_A_ is lower by ∼33 kJ/mol. Assuming that the differences in E_A_ are due to the deletion of a single H-bond, the determined energy differences classify the bond between Trp288 and ECN as a moderately strong intermolecular H-bond, since the dissociation energies of H-bonds in the condensed phase range from 0.8 kJ/mol to 162 kJ/mol [the latter being an exceptionally large value unique for HF_2_^−^ (Steiner, 2002)], which is much larger than the value of 9 kJ/mol for H-bonds in water at 298 K (van der Spoel et al., 2006).

## 4 Conclusion

Since the first high-resolution crystal structures of OCP were published (Kerfeld et al., 2003; Wilson et al., 2010), several studies on OCP have investigated the role of the absolutely conserved Tyr201 and Trp288 residues in the functional activity of this protein (Wilson et al., 2010; Wilson et al., 2011; Maksimov et al., 2016; Kerfeld et al., 2017; Yaroshevich et al., 2021). However, the limited possibilities to substitute aromatic amino acids within the standard amino acid repertoire led to ambiguous results, and overwhelmingly to the loss of the most important property of OCP—its photoactivity. To understand the role of H-bonds, one must be able to control their presence or absence, the most difficult task being neither to disrupt the OCP^O^ form of the protein, nor the ability to photoswitch. These requirements are met by the approach adopted in this work, in which the isosteric exchange of a nitrogen by a sulphur atom in the OCP-W288_BTA variant precisely removes one of the two H-bond donors to the ketocarotenoid.

The X-ray crystal structure clearly reflects the minimal-invasive, hence isomorphous nature of this exchange, and the structural and spectroscopic properties of the OCP^O^ state as well as photoactivity were preserved. Thus, we clearly demonstrate that Trp288 is not *required* for OCP photoactivity. The same is true for Tyr201, as shown by our previous work on the OCP-Y201W variant generated by classical site-directed mutagenesis (Yaroshevich et al., 2021). Thus, it is obvious that the presence of at least one H-bond donor at either of the two sites is *sufficient* to ensure proper formation of the OCP^O^ state and photoactivity. Interestingly, removing one of the two H-bond donors competing for the keto oxygen leads to the intriguing consequence that the carotenoid forms a *more stable interaction* with the remaining H-bond donor. As a result, the protein lacks the spectral heterogeneity characteristic of the dark-adapted state of the wild-type OCP protein.

From the point of view of a protein engineer or a spectroscopist, the spectral homogeneity of the dark-adapted OCP^O^ state and, consequently, the higher yield of the S* photoproduct, which was proposed to be crucial for the productive photocycle (Yaroshevich et al., 2021), could be considered as advantageous evolutionary selection criteria. Therefore, one may speculate why nature solved the problem of designing the OCP photoswitch with two H-bond donors, because obviously the selection criterion was neither the spectral homogeneity of the OCP^O^ ground state nor the quantum efficiency of the photoconversion. Rather, the incorporation of two H-bond donors competing for the ketocarotenoid was a compromise to ensure that the formation of the dark-adapted OCP^O^ state from the highly flexible structural arrangement of the domain-separated OCP^R^ state occurs with high reliability. Apparently, the two competing H-bond donors provide the necessary flexibility or functional redundancy for the compact dark-adapted OCP^O^ state to be formed.

Replacement of Trp288 with BTA reduced the activation energies of photoconversion and recovery by approximately 40 kJ/mol and 33 kJ/mol, respectively, compared with wild-type OCP. Attributing these enthalpy differences to the deletion of a single H-bond, the determined values classify the N-H⋯O = C bond between Trp288 and ECN as a moderately strong intermolecular H-bond. Since all H-bonds can be considered as precursors to proton transfer reactions, which may be well advanced for strong H-bonds (Steiner, 2002), the determined enthalpy differences due to the deletion of an H-bond in OCP are important, especially in view of the recently proposed photoactivation pathway of OCP involving an oxocarbenium ion at the ECN cofactor (Yaroshevich et al., 2021).

Undoubtedly, we now have a much better understanding of the nature of the interactions between the carotenoid ligands and protein matrix of the OCP. Moreover, how the presence of a “surrogate” or “redundant” hydrogen bond may affect the photoactivation process and its biological role has not been studied before. The low quantum yield in the formation of the primary and all subsequent photoproducts in the OCP photocycle (Shirshin et al., 2017) is obviously important for maintaining the balance between photosynthetic activity and photoprotective processes in cyanobacterial cells. We have demonstrated that the use of chemically controlled molecular operations based on the incorporation of non-canonical amino acids offers great potential for better elucidation of protein design by nature, but also for the engineering of such complex and biologically relevant chemical photoswitches in the future.

## Data Availability

The datasets presented in this study can be found in online repositories. The names of the repository/repositories and accession number(s) can be found in the article/Supplementary Material.

## Footnotes

1 to be released upon publication.

## Abbreviations

BTA, 3-benzothienyl-*L*-alanine; CAN, canthaxanthin; ECN, echinenone; ESI-MS, electrospray ionization mass spectrometry; IPTG, isopropyl-β-*D*-thiogalactopyranoside; MmPylRS, pyrrolysine-tRNA synthetase from *Methanosarcina mazei*; ncAA, non-canonical amino acid; OCP, Orange Carotenoid Protein; OCP^O^, dark-adapted orange form of OCP; OCP^R^, light-adapted (active) red form of OCP; PBS, phycobilisome; RR, resonance Raman; SEC, size-exclusion chromatography; Trp, tryptophan; Tyr, tyrosine.
