# Supplementary material for: Parameterization of a single H-bond in Orange Carotenoid Protein by atomic mutation reveals principles of evolutionary design of complex chemical photosystems
Source: Front Mol Biosci. 2023 Jan 26;10:1072606. doi: 10.3389/fmolb.2023.1072606 (PMC9909426; doi:10.3389/fmolb.2023.1072606)
Supplement: Supplementary file 1 [file DataSheet1.pdf]

Supplementary Material

1 Supplementary Figures

1.1 Supplementary Figure S1: Carotenoid content analysis of the OCP-W288_BTA protein

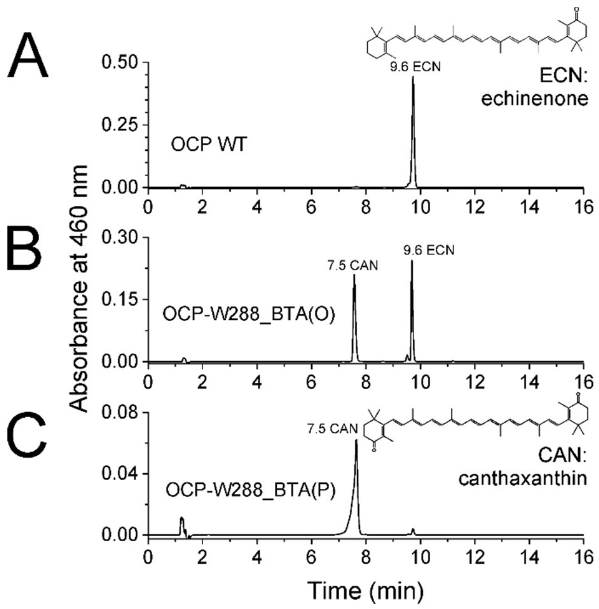

Supplementary Figure S1: Carotenoid content analysis of the OCP-W288_BTA protein. HPLC profiles (absorbance at 460 nm) of carotenoids extracted from wild-type OCP (A), the orange fraction of OCP-W288_BTA (B) and the pink fraction of OCP-W288_BTA (C). The retention times for ECN and CAN are given in each panel. The relative contents of carotenoids are listed in Table 1 of the main article.

1.2 Supplementary Figure S2: Comparison of absorbance spectra of OCP-W288_BTA(P) with other red-shifted OCP holoprotein species.

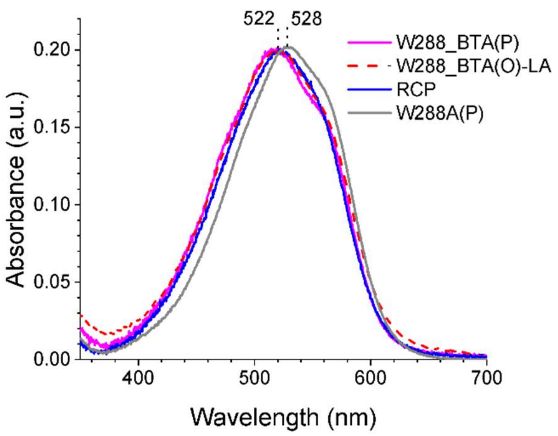

Supplementary Figure S2: Comparison of absorbance spectra of the OCP-W288_BTA(P) protein species with other red-shifted OCP-related holoproteins. The absorbance spectra of the OCP-W288_BTA(P) fraction and the light-adapted OCP-W288_BTA(O) species are shown in comparison with the absorbance spectra of the Red Carotenoid Protein (RCP, i.e., the OCP-NTD holoprotein) (Moldenhauer et al., 2017), and of the pink fraction of the OCP-W288A mutant protein (Maksimov et al., 2016).

1.3 Supplementary Figure S3: Comparison of the *2mFo-DFc* electron density maps around BTA288 in the structure of OCP-W288_BTA (PDB: 7ZXV) and Trp288 in wild-type *Synechocystis* OCP (PDB: 3MG1)

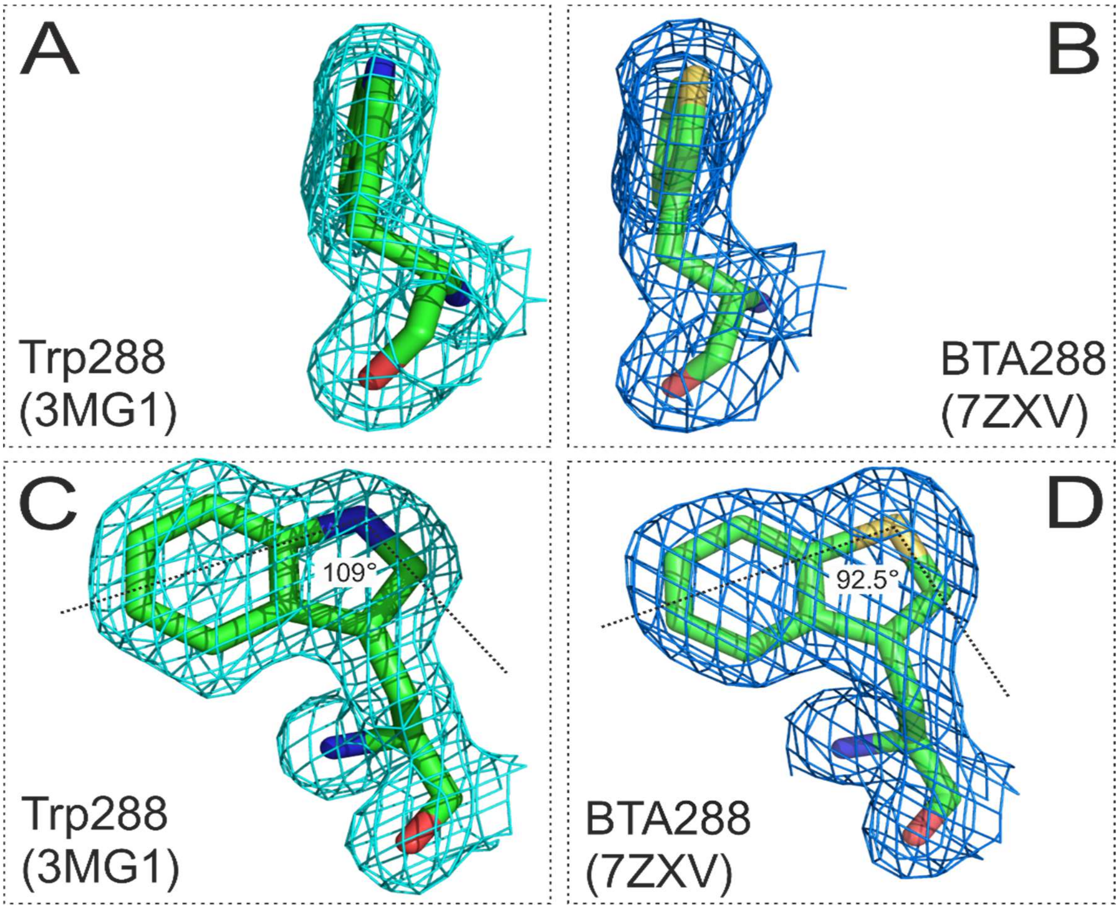

Supplementary Figure S3: Comparison of the configurations of BTA288 in the 7ZXV and Trp288 in the 3MG1 crystal structure. (**A,B**) The *2mFo-DFc* electron density maps along the heterocycle planes of Trp288 (**A**) and BTA288 (**B**) reflect the larger size of the sulphur compared to the nitrogen atom. (**C,D**) Top view of the *2mFo-DFc* electron density maps on the heterocycles of the Trp288 (**C**) and BTA288 (**D**) side chains with the C-N-C angle in (**C**) and the C-S-C angle in (**D**) indicated.

1.4 Supplementary Figure S4: Comparison of structures of OCP-W288_BTA and wild-type *Synechocystis* OCP coordinating CAN (PDB: 4XB5)

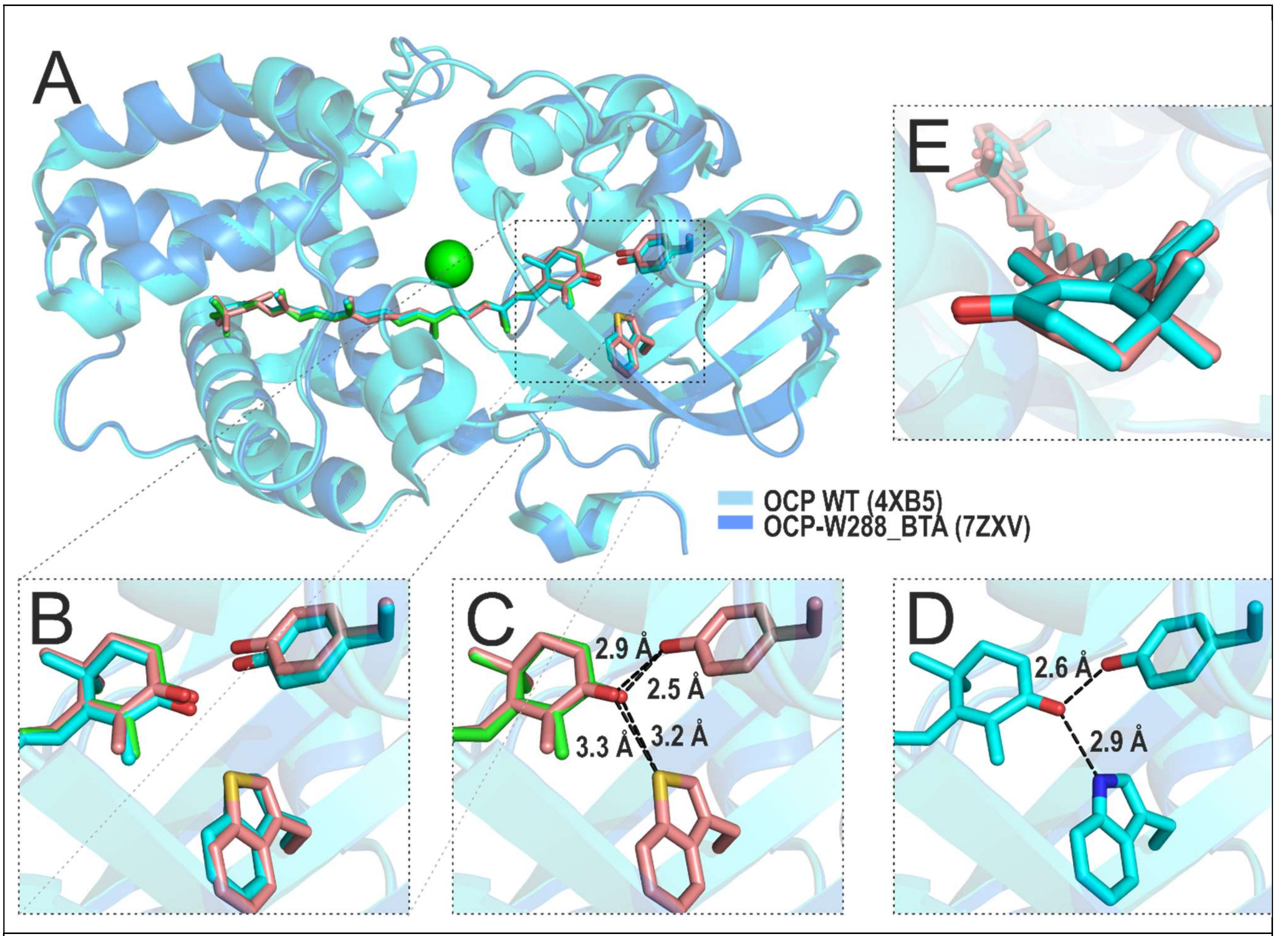

Supplementary Figure S4: Comparison of crystal structures of the OCP-W288_BTA variant (PDB: 7ZXV) with wild-type *Synechocystis* OCP coordinating CAN (PDB: 4XB5). (**A**) The superposition of OCP-W288_BTA (blue) and wild-type OCP (cyan) shows that the structures are very similar, with an RMSD value of 0.226 Å at the Cα-positions. The embedded xanthophylls are also shown with carbon atoms colored in cyan for the CAN molecule in the wild-type OCP structure, and in green or wheat color for the ECN or CAN molecule in the OCP-W288_BTA variant, respectively. Note the almost identical position of the sulphur atom in the BTA residue and the nitrogen atom in Trp288 upon alignment. (**B**) Enlarged view of the boxed area in the CTD from (**A**) with the same color coding. (**C,D**) Details showing the cofactor coordination for OCP-W288_BTA (**C**) and wild-type OCP (**D**). The distance between the sulphur atom in BTA288 and the keto oxygen atom of the xanthophyllic cofactor(s) is slightly increased from 2.9 Å to 3.2 Å (ECN) or 3.3 Å (CAN) compared to the coordination of CAN by Trp288 in wild-type OCP. The distance to the oxygen atom of Tyr201 is comparable to wild-type OCP (2.6 Å) for ECN (2.5 Å), and slightly increased for CAN (2.9 Å). **E**: The configurations of the ketolated end rings of the CAN molecule in wild-type OCP and the OCP-W288_BTA variant in the NTD are almost identical, with a deviation of 0.2 Å at the oxygen atoms.

1.5 Supplementary Figure S5: Results of the 10 ps quantum dynamics (QD) calculations of the ECN-Tyr201-Trp288/BTA complexes

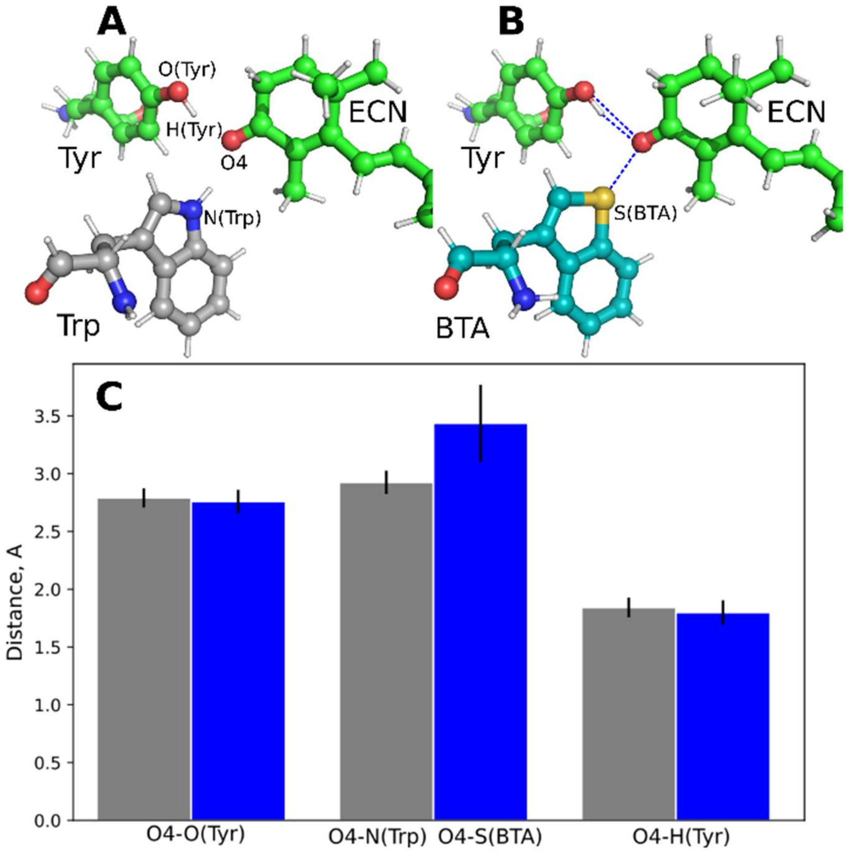

Supplementary Figure S5: Results of the 10 ps quantum dynamics (QD) calculations of the ECN-Tyr201-Trp288/BTA complexes. (**A,B**) Close-up images of the ECN-Tyr201-Trp288 (**A**) and ECN-Tyr201-BTA288 (**B**) complexes. O4 represents the keto oxygen of ECN, O(Tyr) is the oxygen in the hydroxyl group of Tyr201; H(Tyr) is the hydrogen in the hydroxyl group of Tyr201; N(Trp) is the nitrogen of the indole heterocycle in Trp288; S(BTA) represents the sulphur atom in BTA. The main observable distances between atoms are shown as blue dotted lines in **B**. (**C**) The calculated distribution of distances between key atoms in the ECN-Tyr201-Trp288 (gray) and ECN-Tyr201-BTA288 (blue) complexes.

2 References

Maksimov, E. G., Moldenhauer, M., Shirshin, E. A., Parshina, E. A., Sluchanko, N. N., Klementiev, K. E., et al. (2016). A comparative study of three signaling forms of the orange carotenoid protein. *Photosynth. Res.* 130, 389–401. doi: 10.1007/s11120-016-0272-8

Moldenhauer, M., Sluchanko, N. N., Buhrke, D., Zlenko, D. V., Tavraz, N. N., Schmitt, F.-J., et al. (2017). Assembly of photoactive orange carotenoid protein from its domains unravels a carotenoid shuttle mechanism. *Photosynth. Res.* 133, 327–341. doi: 10.1007/s11120-017-0353-3
